# Filter-Free, Harmless, and Single-Wavelength Far UV-C Germicidal Light for Reducing Airborne Pathogenic Viral Infection

**DOI:** 10.3390/v15071463

**Published:** 2023-06-28

**Authors:** Cao-Sang Truong, Palaniyandi Muthukutty, Ho Kyung Jang, Young-Ho Kim, Dong Hoon Lee, So Young Yoo

**Affiliations:** 1 BIO-IT Foundry Technology Institute, Pusan National University, Busan 46241, Republic of Korea; 2 SUNJE HI TEK Co., Ltd., Busan 46047, Republic of Korea; 3 Department of Molecular Biology and Immunology, College of Medicine, Kosin University, Busan 49267, Republic of Korea

**Keywords:** filter-free, harmless, 207 nm, far ultraviolet-C (UV-C), airborne viruses, coronavirus

## Abstract

Germicidal lamps that primarily emit 254 nm ultraviolet (UV) radiation have been effectively utilized for surface sterilization, but they cannot be used on human skin and eyes due to their harmful and genotoxic activity. Recent reports have shown that far UV-C light (207–222 nm) can efficiently kill pathogens with potentially no harm to exposed human tissues. However, these methods still require additional filtering and/or further protective equipment. In this study, we demonstrate a filter-free, harmless, and single-wavelength far UV-C 207 nm germicidal light source that can be used to inactivate different respiratory viruses. It can be exploited as a safe and effective disinfection tool for various airborne viruses. We successfully developed a single-wavelength far UV-C source that produces an exact wavelength of 207 nm. We examined its safety on human skin and corneal cell lines, as well as its effects on inactivating different airborne viruses, such as coronavirus, adenovirus, and vaccinia virus. We expect that our far UV-C lamps can be safely and conveniently used to reduce COVID-19 infections and protect both our living spaces and hospitals from the threat of contamination by possible new or mutant viruses.

## 1. Introduction

Respiratory infections are primarily caused by airborne viruses and can be transmitted through aerosols or contaminated surfaces. Respiratory droplets can vary in size, ranging from submicron levels to approximately 10 μm, with droplets smaller than 5 μm referred to as droplet nuclei [1]. The transmission of respiratory viruses can occur through both airborne droplets and direct contact, resulting in infections in the upper and lower respiratory tracts [2].

One particularly deadly respiratory virus that has recently caused a global pandemic is the severe acute respiratory syndrome coronavirus 2 (SARS-CoV-2). Coronaviruses (CoVs) belong to the Coronaviridae family and are enveloped, single-stranded RNA viruses. They are widely distributed among mammals, including humans. Two types of beta-coronaviruses, namely SARS-CoV and Middle East respiratory syndrome coronavirus (MERS-CoV), emerged in 2003 and 2012, respectively, collectively infecting 10,000 people with mortality rates of 10% and 37% [3,4]. In December 2019, a novel CoV known as SARS-CoV-2 was identified in Wuhan, China, which was responsible for the outbreak of coronavirus disease 2019 (COVID-19).

The COVID-19 pandemic has spread to over 200 countries, resulting in millions of deaths [5], and the surge in COVID-19 infections poses a significant threat to human society. Airborne transmission of diseases has been a public health concern even before the current COVID-19 pandemic. Poorly ventilated rooms or enclosed spaces raise concerns regarding the transmission of respiratory viruses. Wearing masks can prevent transmission from one person to another and minimize the deposition of droplets on surfaces. However, it is important to understand the implications of prolonged mask use in workplaces, social gatherings, and public spaces [6].

Extensive research has been conducted on using natural and mechanical ventilation to control airborne pathogens. Engineering ventilation systems in rooms can help mitigate the spread of pathogens to some extent, although managing transmission through droplets presents greater challenges [7]. Alongside ventilation, the use of room air cleaners and upper-room germicidal fixtures is considered a viable option. However, the effectiveness of room air cleaners in controlling airborne pathogens varies, and their impact is limited. Therefore, ultraviolet (UV)-based irradiation is employed in controlled environments to prevent contamination [8].

UV irradiation has traditionally been a reliable method of disinfection due to its germicidal properties. Germicidal lamps emitting a primarily 254 nm wavelength have proven effective for surface sterilization; however, they are unsuitable for use on human skin and eyes due to their harmful and genotoxic effects. To address the need for air disinfection, UV germicidal irradiation (UVGI) with lower wavelengths can be employed, offering some control over airborne pathogens while remaining energy-efficient and cost-effective [9,10]. UVGI encompasses wavelengths ranging from 200 to 280 nm, whereas far UV-C radiation specifically refers to the lower end of this spectrum, with a range of 200 to 230 nm [11]. The practical production of UV light below 200 nm presents limitations, with the lower limit typically set at 200 nm due to considerations of permeability at atmospheric pressure and the issue of ozone production resulting from oxygen absorption.

In recent times, extensive research has focused on the application of far UV-C radiation within the range of 207–222 nm for controlling viruses and their impact on other biological materials [12,13]. Previous in vitro and in vivo studies have demonstrated the safety of UV-C radiation for humans, as the upper layer of the skin (stratum corneum) and the outer tear layer of the eyes (cornea) provide protection against irradiation. However, airborne pathogens such as bacteria and viruses, lacking these protective layers, are highly vulnerable to UV-C irradiation [14,15,16,17]. Therefore, far UV-C irradiation can be safely used as an effective disinfectant against pathogens in open environments.

In this study, we present our indigenously designed far UV-C irradiation excimer lamps, emitting a single wavelength of 207 nm, which can effectively target viruses without causing harm to human cells. The objective of this research was to assess the impact of far UV-C irradiation on pathogenic respiratory viruses and its potential for controlling their spread in open environments. However, before implementing this technology, its effectiveness and safety in a controlled environment needed to be evaluated.

**Figure 1:**
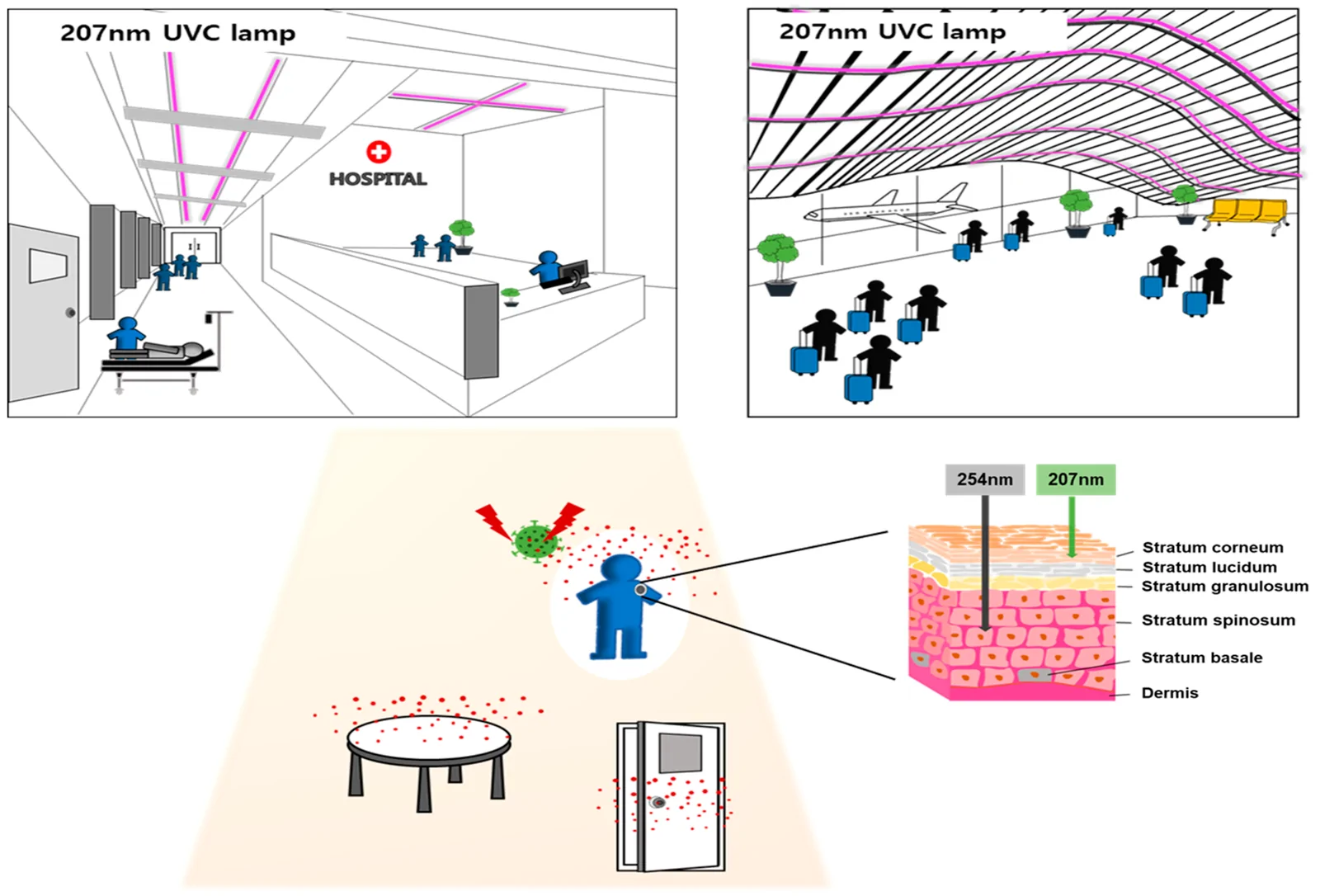
Schematic depicting how our proposed far UV-C device can be applied to living spaces.

Our specially designed far UV-C irradiation module operates by drawing in air and subjecting aerosols to sterilization through irradiation. Subsequently, the released air undergoes a second round of irradiation, resulting in a higher level of sterilization compared to other commercially available devices. We propose the use of this technology as an upper-room UVGI system, suitable for deployment in microbiologically hazardous areas and high-traffic public spaces such as hospitals and airports. By employing this module, we can effectively mitigate the risk of pathogen transmission in the future (Figure 1).

## 2. Materials and Methods

### 2.1. 207 nm Far UV-C Lamp Development and Characterization

A far UV-C single-wavelength excimer lamp (207 nm) module was designed using an optimized inert-gas pressure excimer lamp with a mixture of gases, enabling emission of a specific wavelength. The UV-C irradiation excimer lamp exhibited a single-wave peak at 207 nm and had dimensions of 30 mm in diameter and 310 mm in length.

### 2.2. Cell Lines

MRC5 and Vero cell lines were obtained from the Korean cell line bank located in Seoul, South Korea. HCE-2 and HaCaT cell lines were acquired from the ATCC in Manassas, VA, USA, and CLS in Köln, Germany, respectively. The cells were cultured in Dulbecco’s Modified Eagle Medium (DMEM) supplemented with 10% heat-inactivated fetal bovine serum (FBS) and 1% penicillin/streptomycin (P/S). Cell culture media and reagents were sourced from Welgene, located in Daegu, South Korea. The cells were subcultured and maintained in a 37 °C incubator with 5% CO_2_.

### 2.3. Viral Culture

Human coronavirus HCoV-OC43 (beta-coronavirus 1, VR-1558), vaccinia virus (Wyeth strain, VR-1536), and human adenovirus 5 (Ad5, VR-5) were procured from the ATCC. For HCoV-OC43, viral amplification was performed in Vero cells. Vero cells were infected with the virus and incubated in DMEM supplemented with 2% FBS and 1% P/S. Once complete cytopathic effects (CPEs) were observed, the cell suspension was collected and centrifuged at 117× *g* for 5 min to remove cellular debris. The resulting supernatant was then filtered through a 0.45 µm syringe filter.

VR-1536 and Ad5 were amplified in HeLa cells. These cells were infected with the respective viruses at a multiplicity of infection (MOI) of 0.01 and incubated in DMEM containing 10% FBS and 1% P/S. After the development of complete CPE, the cell suspension was collected in conical tubes and stored in a deep freezer. The freeze/thaw cycle was repeated three times, followed by centrifugation at 1006× *g* at room temperature for 10 min. The resulting supernatant was filtered through 0.45 µm syringe filters. The virus titer was determined using the TCID50 method and expressed as TCID50/mL, as described previously [18].

### 2.4. UV-C Light Exposure to the Virus 

Virus suspensions (100 µL) with varying concentrations for each virus were evenly spread on glass slides and air-dried in a biosafety cabinet at room temperature. Subsequently, the viruses were subjected to UV-C light exposure at different wavelengths (207 nm, 222 nm, and 254 nm) and varied time durations (0 s, 1 s, 10 s, 30 s, 10 min, and 30 min). Following exposure, phosphate-buffered saline solution (PBS) was applied to the glass slides to collect the viruses into microcentrifuge tubes. The experimental procedures were performed in duplicate to ensure reproducibility.

### 2.5. UV-C Light Exposure to Cells

HCE-2 and HaCaT cells were seeded in six-well plates at a density of 3 × 10^5^ cells per well. Following overnight incubation, the cells were subjected to UV-C light exposure using various wavelengths and time periods (energy).

### 2.6. Cell-Viability Assay

The cells were trypsinized, pelleted, and suspended in a mixture of 0.2 mL of medium, 0.5 mL of 0.4% trypan blue solution, and 0.3 mL of PBS. The samples were thoroughly mixed, incubated at room temperature for 15 min, and then examined under a light microscope. A minimum of 300 cells were counted to determine cell survival.

### 2.7. Western Blotting

The cells were lysed using 1× Laemmli lysis buffer (2.4 M glycerol, 0.14 M Tris, pH 6.8, 0.21 M SDS, 0.3 mM bromophenol blue) and then boiled for 10 min. The protein content was measured using a BCA protein assay reagent (Pierce, Rockford, IL, USA). The samples were diluted with 1× Laemmli lysis buffer containing 1.28 M β-mercaptoethanol, and equal amounts of protein were loaded onto 8–12% SDS-polyacrylamide gels. SDS-PAGE was performed to separate the proteins, which were then electrophoretically transferred onto a nitrocellulose membrane. The membrane was blocked with 5% nonfat dry milk in PBS-Tween 20 (0.1%, *v*/*v*) for 1 h. Afterward, the membrane was incubated with a primary antibody (diluted according to the manufacturer’s instructions) at room temperature for 1.5 h. Horseradish peroxidase-conjugated antirabbit or antimouse IgG antibodies were used as secondary antibodies. Immunoreactive protein bands were visualized using the chemiluminescence protocol (ECL, Amersham, Arlington Heights, IL, USA).

## 3. Results 

### 3.1. 207 nm Single-Wavelength Far UV-C Lamp Development

The spectrum of conventional germicidal UV lamps is typically broad, encompassing wavelengths that can be harmful to human health [19]. In this study, we employed a modified lamp resembling a rare-gas electrodeless excimer lamp, using a combination of inert gases at optimized pressure to emit a specific partial wavelength at 207 nm (as described in Table 1).

**Figure 2:**
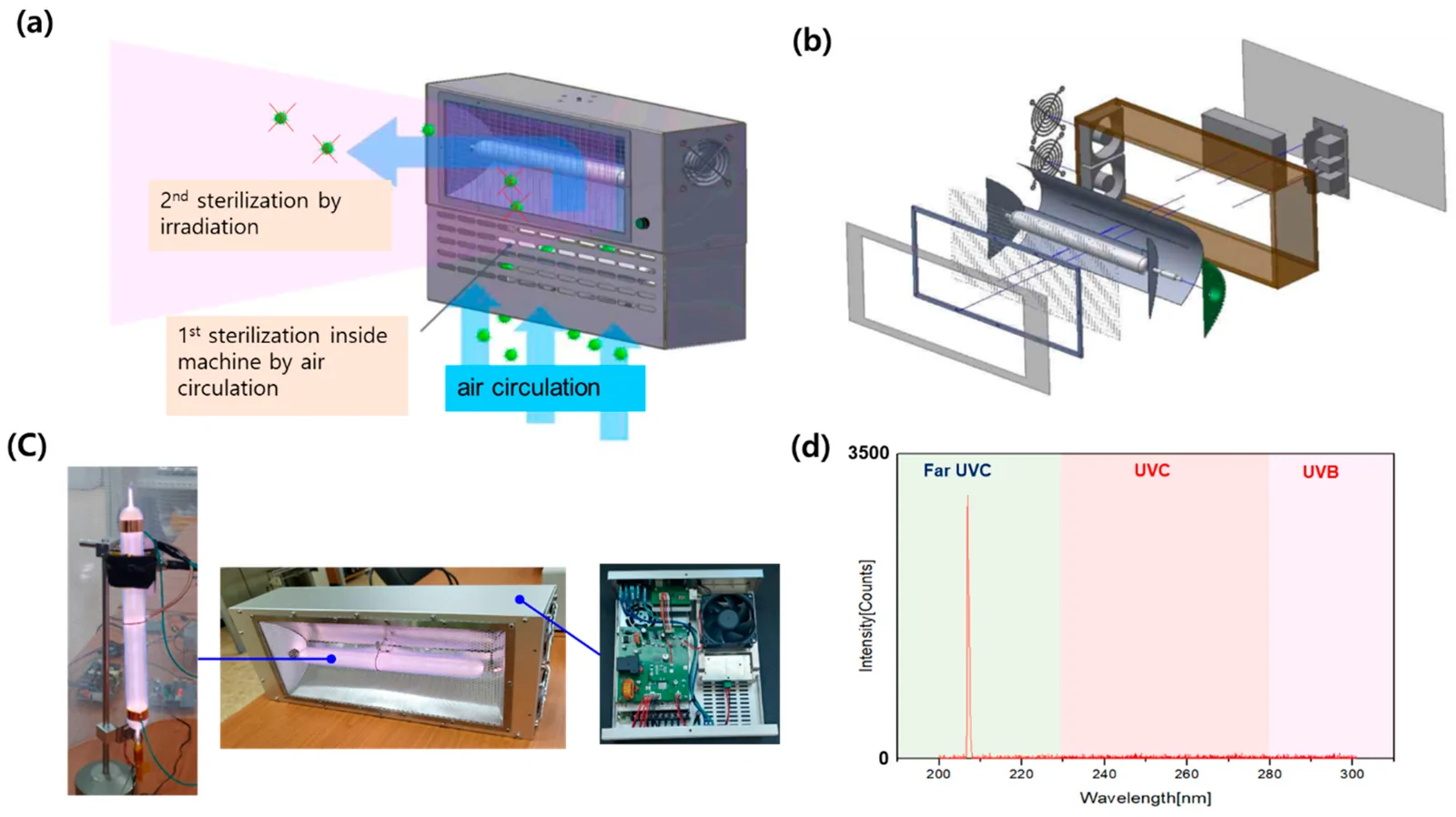
The 207 nm far UV-C lamp development and characterization. (**a**) Our proposed far UV-C device architecture. (**b**) Structure diagram of products (excluding RF power part), focusing on efficiently disinfecting a larger area and being able to sterilize the air in the entire space. (**c**) Photograph of our developed lamp. Photos of the lamp having the spectrum (**left**), the final device format (**middle**), and interior part of the device (**right**). (**d**) The 207 nm single-wavelength peak in spectra power distribution.

Our adapted excimer lamp emitted a single-wavelength peak precisely at 207 nm, effectively minimizing the presence of other wavelengths without the need for optical filtering (Figure 2).

### 3.2. Safety Evaluation of UV-C Light Irradiated onto Human Skin and Corneal Cells

The effectiveness of the proposed lamp’s irradiation needs careful validation to ensure its ability to inactivate pathogens, while also ensuring the safety of UV radiation-based disinfection in enclosed environments. Standardization of UV-C radiation within the range of 200 to 280 nm is necessary to achieve effective pathogen inactivation without compromising human safety. In our study, we compared three different wavelengths within the far UV-C range (207 nm, 222 nm, and 254 nm), considering their photonic effects. Although 254 nm germicidal UV light is highly effective for microbial inactivation, it is not suitable for direct exposure to human skin and eyes because it can induce cytotoxic and DNA-damaging effects in mammalian tissues. Prolonged exposure to this wavelength can result in cytotoxicity and the development of skin malignancies and eye conditions, such as photokeratitis [20,21]. In contrast, far UV-C radiation in the 207–222 nm range demonstrates effective germicidal activity against microorganisms while causing minimal damage to biological materials and humans upon direct exposure.

**Figure 3:**
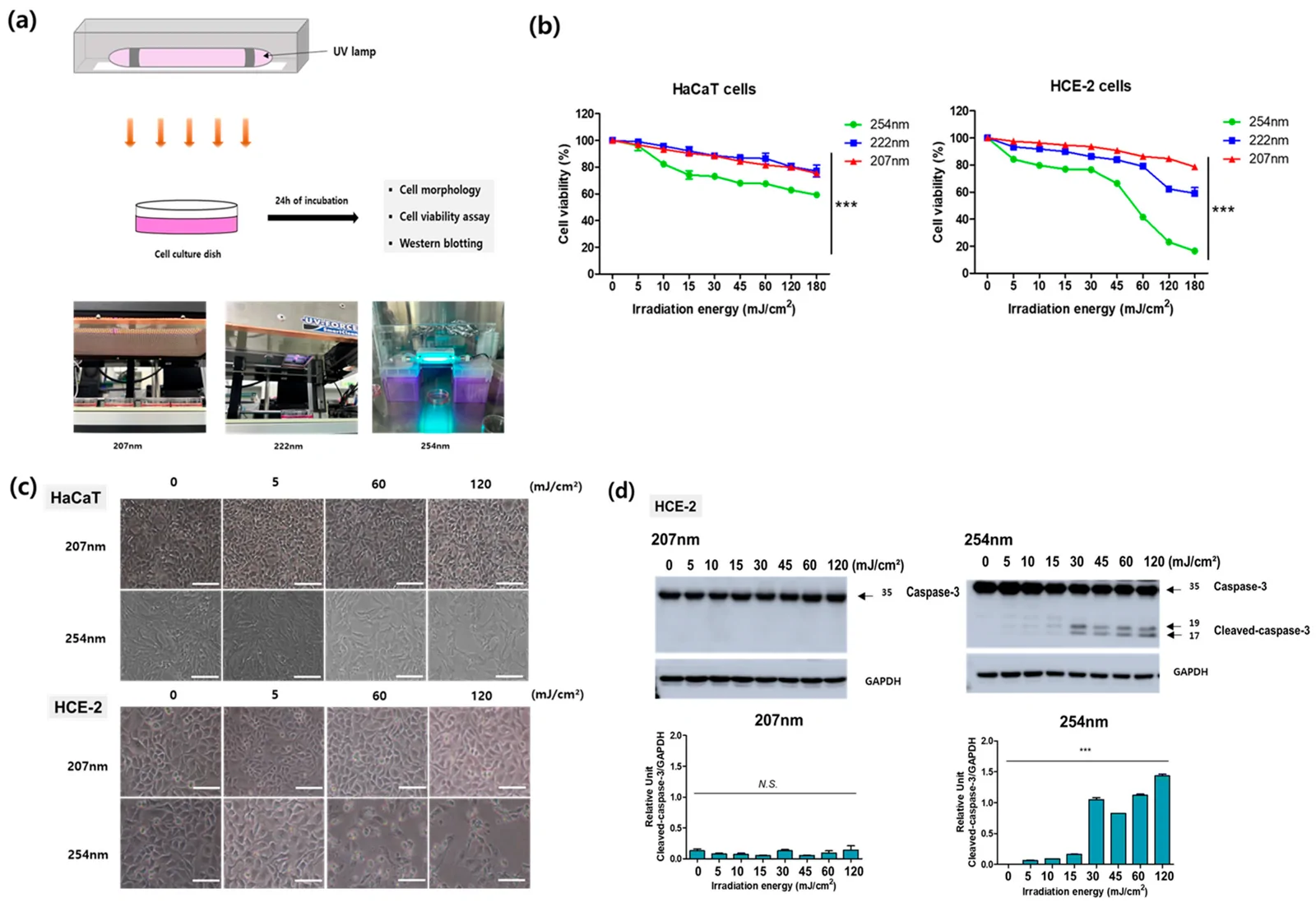
Safety evaluation of 207 nm far UV-C lamp on human skin (HaCaT) and corneal (HCE-2) cell lines. (**a**) Schematic of strategy used to expose cells to UV irradiation and analyze the samples after 24 h. (**b**) Viability (%) of skin (HaCaT) and eye (HCE-2) cells when exposed to three different irradiation wavelengths at increasing energy levels. (**c**) The morphology and cultural properties of HaCaT and HCE-2 cells were compared between 207 and 254 nm wavelengths at various irradiation energies. (**d**) Caspase-3 activation by cleaving to form p19 and p17, which led to apoptosis, confirmed using Western blotting, and quantification after irradiation at 207 and 254 nm on human corneal cells. Two independent experiments were carried out. *N.S*.: not significant (*p* > 0.5); *** *p* > 0.0001, one-way ANOVA.

We investigated to assess the potential cytotoxicity of far UV-C light at different wavelengths in human keratinocyte skin cells (HaCaT cells) and human corneal epithelial cells (HCE-2). The cells were exposed to far UV-C light within an energy range of 0–180 (mJ/cm^2^) and incubated for 24 h. Changes in cell morphology, cytotoxicity, and cleavage of the cell-death-related protein caspase-3 (as an indicator of irradiation-related cell damage) were analyzed through Western blotting of the UV-C light-exposed cells (Figure 3a). We also examined the effective irradiation energy and cell viability loss when the skin and eye cells were exposed to three different far UV-C wavelengths.

According to ISO 10993-5:2009 (Section 8.5), a reduction in cell viability of more than 30% (i.e., viability < 70% relative to the untreated control) is considered indicative of cytotoxicity. In this study, 207 nm far UV-C irradiation resulted in approximately 80% or higher cell viability in HaCaT and HCE-2 cells, which is well above the ISO 10993-5 cytotoxicity threshold. In contrast, 254 nm irradiation caused a substantial decrease in cell viability below this threshold. As depicted in Figure 3b, HaCaT cells demonstrated higher mortality (approximately 60%) when exposed to 254 nm, whereas irradiation at 222 nm and 207 nm resulted in 80% cell viability even at an energy of 180 mJ/cm^2^. On the other hand, HCE-2 cells exhibited a sharp decrease in viability to less than 20% when exposed to the higher wavelength of 254 nm. However, they showed moderate cell death at 222 nm and the lowest cell viability loss at 207 nm.

We also investigated the effects of irradiation on the morphology and cultural characteristics of the skin and eye cells at 207 nm and 254 nm wavelengths. The results demonstrated that the 207 nm radiation spectra did not have a significant impact on the cells, even at high irradiation energies. In contrast, the 254 nm radiation caused deterioration in both skin and eye cells (Figure 3c). The exposure to 254 nm reduced the cell count, indicating their susceptibility to the radiation effects. Furthermore, cells exposed to 254 nm displayed a more elongated and spindle-like shape compared to those exposed to 207 nm, suggesting that far UV-C irradiation at 254 nm is cytotoxic and induces cell death at higher irradiation energies.

Irradiation can have biological effects on cells, potentially leading to DNA damage, abnormal protein function, and cell death. To examine the impact of irradiation on cells, we detected the cleavage of caspase-3 using Western blotting. Caspase-3 is a crucial molecule involved in the execution of apoptosis. Activation of caspase-3 occurs when its inactive form is cleaved into p19 and p17 fragments, indicating the occurrence of apoptosis. In this study, we aimed to assess the susceptibility of HCE-2 cells to UV-C radiation.

Western blotting analysis revealed that the 207 nm wavelength did not affect the cells. No cleavage of caspase-3 was observed, indicating the absence of apoptosis and cell death. However, at the 254 nm wavelength, increasing irradiation energies led to the cleavage of the pro-caspase-3 protein and its activation into p19 and p17 subunits, resulting in the induction of apoptosis by active caspase-3 (Figure 3d).

### 3.3. Evaluation of UV-C Light against Viral Pathogens

We investigated to determine the rate at which different far UV-C wavelengths could inactivate viruses. Specifically, we exposed coronavirus (OC43), adenovirus (Ad5), and vaccinia virus (VR-1536) to irradiation from a far UV-C excimer lamp source and examined the energy (mJ/cm^2^) required for virus disinfection. The plaque-forming units (PFU/mL) were determined using the median tissue culture infectious dose (TCID50) assay, which measures the amount of virus needed to cause a cytopathic effect in 50% of infected cells [22].

**Figure 4:**
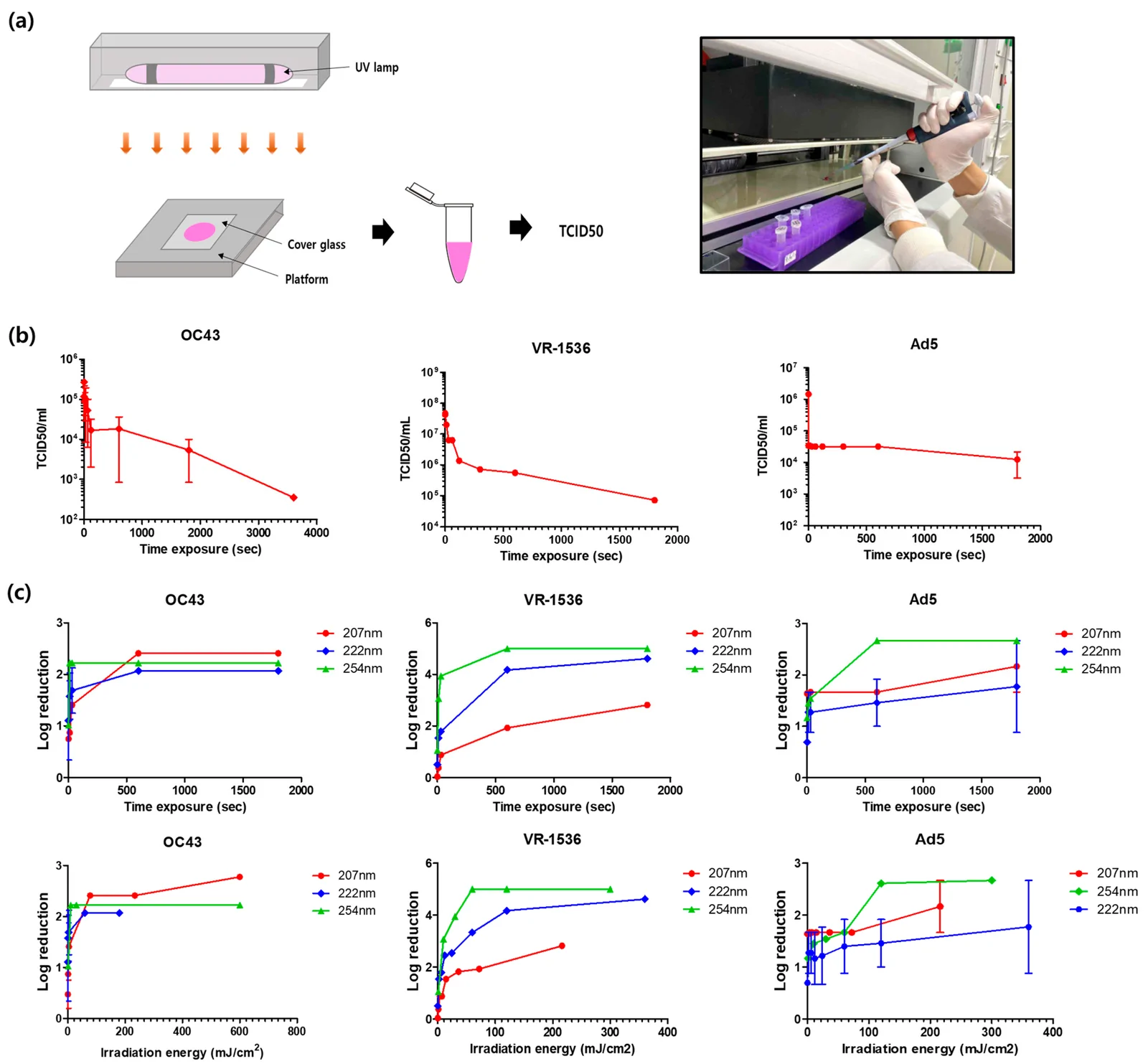
Effectiveness of 207 nm far UV-C lamp in inactivating different types of viruses compared to those of the 222 nm and 254 nm lamps. (**a**) Schematic of the setup required for irradiating viruses under three different UV wavelengths. (**b**) TCID_50_/mL for coronavirus (OC43), vaccinia virus (VR-1536), and adenovirus (Ad5) after irradiation at different time exposures at 207 nm. (**c**) Log reduction showing the efficiency of the 207, 222, and 254 nm wavelength lamps against OC43, VR-1536, and Ad5 at different time exposures (**up**) and different irradiation energy levels (**down**). All graph data are shown with mean ± SEM. Three to four independent experiments were performed with OC43 at 207 and 222 nm. Two experiments were performed with the others.

The viruses were exposed to three different UV-C wavelength spectra (207, 222, and 254 nm) to analyze their irradiation capacities, and their log reductions were calculated. All three virus samples were loaded onto slides, dried, and exposed to irradiation for different time durations. Figure 4a illustrates the experimental setup by which the viruses were exposed to irradiation. All three exposed viruses were quantified using TCID_50_ at different time exposures to determine if the viruses were effectively denatured.

Figure 4b displays the log reduction in TCID50, demonstrating that the disinfection rate increased over time for all three viruses. OC43 exposed to the 207 nm far UV-C spectrum exhibited 82% inactivation within 1 s and 99% inactivation within 10 min at an emitted far UV-C dose of 78 mJ/cm^2^. No significant variations were observed when the same treatment was extended to 30 min (Table 2).

To quantify virus disinfection through radiation energy, we exposed the three different viruses to three different wavelengths of irradiation and evaluated the log reduction. Figure 4c illustrates that a 1 log reduction in OC43 required 1.3 mJ/cm^2^ of 207 nm light and 1 mJ/cm^2^ of 254 nm light. For the 222 nm light source, inactivation efficiency is presented as a function of exposure time, as directly comparable energy values could not be determined under the same measurement conditions. VR-1536 exhibited better inactivation with higher log reductions at 207 nm, and a similar trend was observed for the 222 nm and 254 nm sources. However, Ad5 showed better inactivation with the 254 nm source, followed by the 222 nm and, lastly, the 207 nm sources.

The 207 nm light source demonstrated superior inactivation and log reduction for OC43 due to its higher kilo base pairs compared to other ssRNA viruses. Similarly, VR-1536, which has bulk dsDNA, showed similar trends, indicating a higher probability of inactivation through the formation of cyclobutene pyrimidine dimers (CPDs). As a result, less irradiation energy is required to achieve 99% inactivation in a shorter time period. The 222 nm source also exhibited virus inactivation potential, but it necessitates optical filtering to protect users from secondary radiation peaks.

## 4. Discussion

Due to the recent outbreak of the SARS-CoV2 pandemic and the impact of various respiratory diseases on society, there is a need for an effective method to control the spread of germs in the environment. Disinfection and decontamination of environmental pathogens play a crucial role in preventing disease transmission. Zhang et al. monitored SARS-CoV-2 in air and on surfaces and estimated the infection risk [24]. They measured viral amounts with qPCR: 2.3~7.6 × 10^−2^ genome copy/L in air and 6.78 × 10^−3^~1.12 × 10^−2^ genome copy/L on surfaces; 0.023 gc/L of SARS-CoV-2 in the air take 5–15 min to infect 15 out of 100,000 maskless passengers on the bus. Probability of infection increases with the exposure time. Mechanical or natural methods have limitations in controlling airborne transmission, but the use of safe and effective irradiation-based disinfection has shown potential in containing the transmission of viruses [9,25,26]. UV irradiation has been widely used as a germicide in hospitals and high-sterile zones to combat microbial contamination [27]. Among the UV spectrum, UV-A, UV-B, and UV-C have been extensively studied for their efficacy in pathogen control. However, the practical application of irradiation sterilization in open environments has been limited due to the harmful biological effects of UV-A, UV-B, and UV-C on human beings [28]. 

The utilization of germicidal UV lamps for effective control of virus transmission relies on the specific type of lamp used [29]. Mercury-based lamps have traditionally been used to generate germicidal UV-A irradiation, but concerns regarding their potential harm to humans have prompted the exploration of alternative irradiation sources. Excimer, LED, ozone, and xenon lamps have been developed, each with its own set of advantages and disadvantages in terms of modality and efficiency [11]. In comparison to UV-C-emitting mercury lamps, which emit higher-energy radiation, quasimonochromatic excimer lamps that emit high-energy photons in the far UV-C region offer a disinfection capability against pathogenic microorganisms while ensuring human safety [30]. In this study, we designed an irradiation module using an excimer lamp source, capable of generating germicidal 207 nm far UV-C irradiation to effectively neutralize aerosolized viruses.

We investigated the efficacy of different UV-C doses from various lamps in neutralizing viruses using far UV-C germicidal irradiation. The disinfection rates of each lamp were assessed, and results showed that the 254 nm lamp exhibited the highest germicidal capacity, followed by the 207 nm lamp, for OC43. Regarding VR-1536, the 207 nm lamp demonstrated greater inactivation compared to the 222 nm and 254 nm lamps. Our findings align with previous studies conducted by McDevitt et al., which used UV-C irradiation at 254 nm, confirming the efficacy of the 207 nm far UV-C light for controlling aerosolized VR-1536 [31]. Ad5, known for its high resistance to UV radiation among enteric viruses, required 400 J/cm2 irradiation for a 1-log reduction (90% inactivation) in disinfection rates [32]. Consistent with this, our results indicated that Ad5 exhibited higher inactivation rates at 222 nm, followed by 254 nm and 207 nm. Additionally, the size and genetic composition of viruses play a role in their susceptibility to disinfection. Studies have shown that viruses with larger genomes are more susceptible to irradiation, as they provide more targets for the photochemical damage to take effect more rapidly [33]. In line with these findings, our data indicated that VR-1536, which contains dsDNA (~190 kbp), experienced significantly higher inactivation compared to Ad5 (~35 kbp). OC43 (~30 kbp), with the largest known genome among ssRNA viruses, was effectively inactivated by the 207 nm lamp. The reduction in 2–3 logs is considered a substantial amount of virus to have been reduced, which matches 99–99.9% inactivation. The data show the overall reduction in virus load. Thus, our study demonstrates that the 207 nm lamp can effectively neutralize most viruses without causing photoirradiation effects on humans.

According to the guidelines set by the International Commission on Non-Ionizing Radiation Protection (ICNIRP), the exposure limits for UV irradiation should not exceed 30 J/cm^2^ for skin and eye exposure [34]. For far UV-C irradiation at 207 nm, the permissible exposure limit is 51.4 mJ/cm^2^, with a maximum of 8 mJ/cm^2^ per 8 h exposure [30,34]. Our findings demonstrate that exposure of OC43 to far UV-C irradiation resulted in 96% viral inactivation (3.9 mJ/cm^2^) within 30 s and 99% inactivation within 10 min, well within the regulatory limits defined by the guidelines. Additionally, doses ranging from 1 s (0.13 mJ/cm^2^) to 10 s (1.3 mJ/cm^2^) achieved effective disinfection rates of 82% and 86%, respectively. These results highlight that OC43 can be rapidly neutralized upon exposure to far UV-C irradiation at 207 nm, providing an efficient approach to mitigate airborne respiratory viruses in public settings.

Humans are exposed to UV irradiation from solar radiation, which can have harmful biological effects and increase the risk of cancer, including melanoma and non-melanoma skin cancers. Consequently, concerns about the safety of using UV irradiation as a disinfectant have arisen, particularly regarding the spectra that are implicated in photocarcinogenesis, such as UV-A and UV-B [35]. In the case of UV-C, the use of 254 nm light for germicidal purposes is common, but it can penetrate the basal layer and cause erythema. However, recent studies have highlighted the unique properties of far UV-C radiation around 200 nm. This type of radiation has poor penetration into biological materials and is readily absorbed by proteins, peptide bonds, and other biomolecules [36]. Mammalian cells, compared to bacteria and viruses, are larger in size and can absorb around 95% of the irradiation on their surface. Consequently, UV-C irradiation at 200 nm is unable to penetrate the cells and cause damage to their nuclei [37,38,39]. Furthermore, even prolonged exposure to UV-C irradiation in the 200 nm range does not harm cells or organs, as most of the irradiation is absorbed by the cell membrane itself [40,41].

Studies have been conducted on cell lines and animal models to investigate the mechanism of interaction and determine the threshold of irradiation at which biological effects, such as minor skin reactions and carcinogenic potential, manifest through CPD markers [39,42]. Furthermore, extensive research has been carried out on the effectiveness of the 222 nm wavelength range in controlling alpha and beta coronaviruses and influenza viruses, as well as determining the threshold in human cells [43,44]. In addition, Buonanno et al. demonstrated the efficacy of an excimer lamp emitting 207 nm far UV-C in vitro for controlling bacterial infections. Moreover, studies using mouse models have shown that the germicidal effect of the 207 nm wavelength is comparable to the 254 nm range, without causing harm to human skin cells [14,15]. Since the aim of this study is to use in an upper-room UVGI, exposure to long durations can be potentially a hazard, causing dermatitis and increased chance of skin cancer. Thus, single far UV-C irradiation using 207 nm with our device is potentially safe and effective for controlling airborne pathogens [29,45].

A recent proof-of-concept skin-testing case study conducted by Eadie et al. demonstrated that using a 222 nm wavelength of far UV-C up to 1500 mJ/cm^2^ did not cause any visible changes to the skin. However, a dose of 6000 mJ/cm^2^ resulted in yellow coloring of the skin without causing erythema. In contrast, achieving a 99% inactivation of OC43 required only 78 mJ/cm^2^ for a duration of 10 min [46]. Another study by Kaidzu et al. investigated rat corneal keratitis and found that the minimal threshold dose for 207 nm was 15,000 mJ/cm^2^. Histological analysis revealed that CPD damage occurred only in the superficial layers of the cornea. In contrast, exposure to 254 nm UV-C irradiation strongly affected the limbus and cornea, leading to CPD formation in these layers [47]. Our study provides an innovative in vitro model using skin and eye cells, demonstrating the comparative advantage of using 207 nm light in the far UV-C spectrum without any adverse effects on human cells. 

## 5. Conclusions

Finding a solution to the COVID-19 pandemic, as well as addressing its impact on social life and the economy, is of utmost importance. Extensive research and development have been focused on germicidal disinfection and irradiation techniques to control infectious viruses. In this study, we used far UV-C irradiation at a wavelength of 207 nm, which demonstrated efficient disinfection by achieving a 96% inactivation of OC43 in just 10 s at 1.3 mJ/cm^2^. Importantly, our findings also showed that far UV-C irradiation at 207 nm does not cause harm to either skin and corneal cells, even at a higher dose of 120 mJ/cm^2^. Furthermore, compared to studies using the existing 254 nm UV-C irradiation wavelength, our study minimized cytotoxicity to cells by reducing the activation of apoptosis.

In summary, our study suggests that far UV-C at 207 nm is an effective germicide while posing no harm to human cells. These results validate the viability of this technology as a safe strategy for decontamination in public places, enabling effective control of respiratory viruses and airborne infections. 

## Figures and Tables

**Table 1:** 207 nm far UV-C lamp information.

Aperture Size	Peak Wavelength	Input Power	Consumption Power	Weight	Lamp Lifer Time
368 × 150 (mm)	Far UVC 207 nm	AC100~AC240 V	80 w/h	3.8 kg	3000 h

**Table 2:** Effectiveness of 207 nm UV-C light on the disinfection of human coronavirus HCoV-OC43.

Time Exposure	Control	1 s	10 s	30 s	10 min	30 min
Energy (mJ/cm^2^)	0	0.13	1.3	3.9	78	234
TCID50/mL	2.15 × 10^5^	3.86 × 10^4^	2.94 × 10^4^	8.43 × 10^3^	8.43 × 10^2^ *	8.43 × 10^2^ *
Log reduction	0	0.75	0.87	1.41	2.41	2.41
Inactivation (%)	0	82	86	96	99	99

* The reason for the absence of a decrease in viral titer greater than 78 mJ/cm^2^ is due to limitation of viral dilution, which started from 10^−3^. We cannot calculate virus titers below 10^2^ even though it would surely have reduced the viral load [23]. In this case, TCID50 value was calculated by setting the value at 10^−3^ dilution as 100% CPE.

## Data Availability

The data of this study are available from the corresponding authors on reasonable request.
