# Correction: Truong et al. Filter-Free, Harmless, and Single-Wavelength Far UV-C Germicidal Light for Reducing Airborne Pathogenic Viral Infection. *Viruses* 2023, *15*, 1463

**DOI:** 10.3390/v18080824

**Published:** 2026-07-27

**Authors:** Cao-Sang Truong, Palaniyandi Muthukutty, Ho Kyung Jang, Young-Ho Kim, Dong Hoon Lee, So Young Yoo

**Affiliations:** 1 BIO-IT Foundry Technology Institute, Pusan National University, Busan 46241, Republic of Korea; 2 SUNJE HI TEK Co., Ltd., Busan 46047, Republic of Korea; 3 Department of Molecular Biology and Immunology, College of Medicine, Kosin University, Busan 49267, Republic of Korea

**Text Correction**

In the published article [1], the authors would like to make the following corrections to improve clarity, accuracy, and reproducibility.

Correction

3. Results, (3.2.) Safety Evaluation of UV-C Light Irradiated onto Human Skin and Corneal Cells (Para: 1)

Original sentence:

“Although the 254 nm wavelength germicidal light is well known for its efficacy in treating pressure ulcer infections, it can have adverse effects on microorganisms”.

Revised sentence:

“Although 254 nm germicidal UV light is highly effective for microbial inactivation, it is not suitable for direct exposure to human skin and eyes because it can induce cytotoxic and DNA-damaging effects in mammalian tissues”.

2. Clarification

3. Results, (3.2.) Safety Evaluation of UV-C Light Irradiated onto Human Skin and Corneal Cells (Para: 3)

Original sentence:

“The direct exposure on cells might induce a considerable amount of loss of cells within a significant percentage compared to the exposure on the tissues. Therefore, up to 20% cell loss is generally considered as a safety tolerance.” and “In summary, the 254 nm spectra induced cytotoxicity in corneal cells, with the severity increasing as the energy value rose. In contrast, the cytotoxic effects were insignificant for the 207 nm wavelength spectra, even at an energy of 120 mJ/cm^2^”.

We have clarified the interpretation of the cell viability results in the context of the international standard for in vitro cytotoxicity testing.

Revised text:

“According to ISO 10993-5:2009 (Section 8.5), a reduction in cell viability of more than 30% (i.e., viability < 70% relative to the untreated control) is considered indicative of cytotoxicity. In this study, 207 nm far UV-C irradiation resulted in approximately 80% or higher cell viability in HaCaT and HCE-2 cells, which is well above the ISO 10993-5 cytotoxicity threshold. In contrast, 254 nm irradiation caused a substantial decrease in cell viability below this threshold”.

3. Correction and Clarification

3. Results, (3.3.) Safety Evaluation of UV-C Light Irradiated onto Human Skin and Corneal Cells (Figure 4c) (Para: 4)

Original sentence:

“Figure 4c illustrates that a 1 log reduction in OC43 required 1.3 mJ/cm^2^ of 207 nm light, whereas 1 and 1.6 log reductions were necessary for 254 nm (1 mJ/cm^2^) and 222 nm (mJ/cm^2^) light sources, respectively”.

Revised sentence:

“Figure 4c illustrates that a 1 log reduction in OC43 required 1.3 mJ/cm^2^ of 207 nm light and 1 mJ/cm^2^ of 254 nm light. For the 222 nm light source, inactivation efficiency is presented as a function of exposure time, as directly comparable energy values could not be determined under the same measurement conditions”.

4. Correction

4. Discussion (Para: 1)

Original sentence:

“0.023 gc/L of SARS-CoV-2 in the air take 5–15 min to infect out of 100,000 maskless passengers on the bus”.

Revised sentence:

“0.023 gc/L of SARS-CoV-2 in the air take 5–15 min to infect 15 out of 100,000 maskless passengers on the bus”.

5. Correction throughout the manuscript

The company name has been standardized to “SUNJE HI TEK Co., Ltd.” wherever it appeared.

The authors state that the scientific conclusions are unaffected. This correction was approved by the Academic Editor. The original publication has also been updated.
